# Diversity, Phylogeny and Plant Growth Promotion Traits of Nodule Associated Bacteria Isolated from *Lotus parviflorus*

**DOI:** 10.3390/microorganisms8040499

**Published:** 2020-03-31

**Authors:** Ricardo Soares, Jesús Trejo, Maria J. Lorite, Etelvina Figueira, Juan Sanjuán, Isabel Videira e Castro

**Affiliations:** 1 Laboratório de Microbiologia do Solo, UEISSAFSV, Instituto Nacional de Investigação Agrária e Veterinária, I.P. (INIAV, I.P.), 2780-159 Oeiras, Portugal; ricardo.soares@iniav.pt (R.S.); jesusmt26@gmail.com (J.T.); 2 Laboratório de Bioquímica Inorgânica e RMN, Instituto de Tecnologia Química e Biológica António Xavier, Universidade Nova de Lisboa, 2780-157 Oeiras, Portugal; 3 Departamento de Microbiología del Suelo y Sistemas Simbióticos, Estación Experimental del Zaidín, CSIC, E-18160 Granada, Spain; mjlorite@eez.csic.es (M.L.); juan.sanjuan@eez.csic.es (J.S.); 4 Departamento de Biologia & CESAM, Universidade de Aveiro, 3810-193 Aveiro, Portugal; efigueira@ua.pt

**Keywords:** diversity, rhizobia, endophytes, plant growth promoting rhizobacteria (PGPR), *Bradyrhizobium*, *Lotus parviflorus*

## Abstract

*Lotus* spp. are widely used as a forage to improve pastures, and inoculation with elite rhizobial strains is a common practice in many countries. However, only a few *Lotus* species have been studied in the context of plant-rhizobia interactions. In this study, forty highly diverse bacterial strains were isolated from root nodules of wild *Lotus parviflorus* plants growing in two field locations in Portugal. However, only 10% of these isolates could nodulate one or more legume hosts tested, whereas 90% were thought to be opportunistic nodule associated bacteria. Phylogenetic studies place the nodulating isolates within the *Bradyrhizobium* genus, which is closely related to *B. canariense* and other *Bradyrhizobium* sp. strains isolated from genistoid legumes and *Ornithopus* spp. Symbiotic *nodC* and *nifH* gene phylogenies were fully consistent with the taxonomic assignment and host range. The non-nodulating bacteria isolated were alpha- (*Rhizobium/Agrobacterium*), beta- (*Massilia*) and gamma-proteobacteria (*Pseudomonas*, *Lysobacter, Luteibacter*, *Stenotrophomonas* and *Rahnella*), as well as some bacteroidetes from genera *Sphingobacterium* and *Mucilaginibacte*r. Some of these nodule-associated bacteria expressed plant growth promotion (PGP) traits, such as production of lytic enzymes, antagonistic activity against phytopathogens, phosphate solubilization, or siderophore production. This argues for a potential beneficial role of these *L. parviflorus* nodule-associated bacteria.

## 1. Introduction

*Lotus* is the largest genus of the *Loteae* tribe, which includes more than 180 species of perennial and annual herbs and small shrubs distributed in the Mediterranean region and in the Western USA [1,2]. Several *Lotus* spp. (*Lotus uliginosus*, *Lotus corniculatus*, *Lotus tenuis,* and *Lotus subbiflorus*) have forage value and are widely used to improve pastures [2]. Moreover, *L. japonicus* has been chosen as a model legume for genetic studies [3,4]. Like most legumes, *Lotus* spp. can establish nitrogen-fixing symbioses with certain soil bacteria collectively known as rhizobia. Typically, *Lotus* can either form specific interactions with the intermediate growing *Mesorhizobium* genus (group I: *L. corniculatus* and *L. tenuis*, e.g., [5,6,7]) or with the slow growing, *Bradyrhizobium* genus (group II: *L. uliginosus* (*Lotus pedunculatus*)*, Lotus angustissimus* and *L. subbiflorus*, e.g., [8,9,10]). Nevertheless, *Lotus*-rhizobia interactions seem to be more complex, as *Lotus* from group I have been reported to interact with rhizobia belonging to the *Rhizobium* or *Aminobacter* genera [11]. Additionally, it has been found that certain non-cultivated *Lotus* species interact with rhizobia of the genus *Ensifer* [12,13,14]. Therefore, it seems that a lot is to be discovered about *Lotus* root nodule microsymbionts, especially regarding uncultivated species [15].

Genistoid legumes (e.g., *Lupinus* spp., *Cytisus* spp, *Adenocarpus* spp., *Genista* spp., *Spartium* ssp, etc.), Serradella (*Ornithopus* spp.), and *L. uliginosus* have been shown to be nodulated by closely related bradyrhizobia that are in the same cross-inoculation group [9,16,17]. The genus *Bradyrhizobium* comprises highly diversified bacteria, with 39 described species thus far [18,19,20], including some photosynthetic strains [21,22,23]. The 16S rRNA gene is very conserved within this genus and, like the symbiotic genes, has been shown to be laterally transmitted [24,25]. Thus, for a better phylogenetic prediction, multilocus sequence analysis (MLSA) of protein encoding housekeeping genes, such as *rec*A, *atp*D and *gln*II, is more suitable [16,24,25,26].

The legume root nodules have been traditionally considered to be exclusively colonized by rhizobial bacteria, but this view has rapidly changed over the past several years. In addition to rhizobia, other bacteria can frequently inhabit the interior of nodules, e.g., *Bacillus*, *Pseudomonas, Agrobacterium,* and others [27,28,29,30,31,32,33], without causing any damage or disease, and are thus considered nodule endophytic bacteria. These nodule inhabitants are not able to nodulate by themselves and therefore could be viewed as opportunistic colonizers [34]. Recently, it has been reported that *L. japonicus* can control infection of nodules by both the symbiotic and other endophytic bacteria [34]. These findings have given a new perspective for these endophytic bacteria, originally treated as “contaminants”. The role of these non-rhizobia nodule bacteria is still unclear, but some studies point out a possible beneficial role, e.g., enhancing nodulation and nitrogen fixation [35,36,37]. Moreover, besides nitrogen fixation, plant growth can be enhanced through other mechanisms by rhizobia and non-rhizobial bacteria, generally known as plant growth promoting rhizobacteria (PGPR) [38] or plant probiotic bacteria (PPB). Among these other mechanisms are solubilization of mineral nutrients [39,40,41], production of phytohormones [42,43,44], or the expression of antagonistic activities against phytopathogens [45,46,47].

The aim of this work was to identify and characterize bacteria inhabiting the interior of root nodules of field-grown *Lotus parviflorus* plants. To our knowledge, this is the first report on bacteria isolated from root nodules from this legume.

## 2. Materials and Methods

### 2.1. Isolation of Bacterial Strains and Growth Conditions

**Figure 1:**
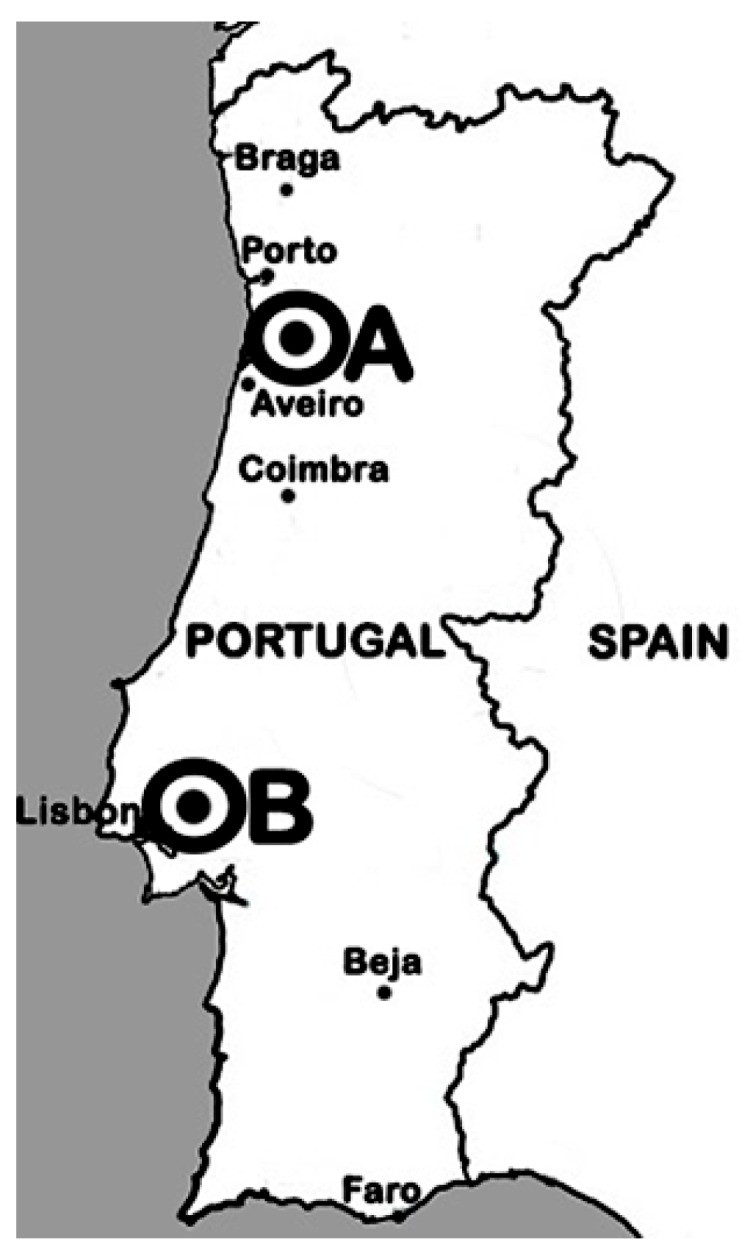
Map location of the sampling sites (A and B).

Bacteria were isolated from effective root nodules of wild *L. parviflorus* plants growing in two field sites in Portugal: Beduído, which belongs to Estarreja city (site A; 40°46′0.064″ N 8° 35′12.035″ W); and fields of the “Companhia das Lezírias” farming exploration company, located in Samora Correia, which belongs to Benavente city (site B; 38° 49′24.7″ N 8°50′36.8″ W; Figure 1). Nodules were surface sterilized [9] then individually crushed before a droplet was spread on a plate with yeast-mannitol agar (YMA) supplemented with congo red dye according to [48,49]. Plates were incubated at 28 °C in the dark for 1 week. The isolate’s purity was checked by examining colony morphology and congo red absorption. Sub-culturing was conducted when more than one type of colony was present. Each pure isolate was stored at 4 °C and at −80 °C with 20% of glycerol.

Absorption of congo red was visualized by growing bacteria in YMA supplemented with 0.25 mg L^−1^ congo red. Congo red dye is often used to distinguish rhizobia from non-rhizobial strains. *Rhizobium* colonies typically do not absorb congo red [48,49], while other bacteria absorb it.

### 2.2. Repetitive Extragenic Palindromic (REP)-PCR Amplification and Fingerprint Analysis

Genomic DNA extractions were done following the procedure by Estrella et al. [11] or using “Aqua pure Genomic DNA extraction” kit from Bio-Rad, following the kit protocol specifications. After DNA extraction, genomic repetitive extragenic palindromic (REP) fingerprints were generated using REP1R and REP2I primers, as previously described by Versalovic, Koeuth, and Lupski [50]. Computer-assisted analysis of the REP fingerprints was performed using GelJ v2.0 software [51]. A dendrogram was constructed using dice coefficient and the unweighted pair group method with arithmetic mean method (UPGMA) clustering method [52] at a 1% band-position tolerance level.

### 2.3. PCR Gene Amplification and Sequencing

16S rRNA gene amplification was performed using 41F and 1488R primers as described by [53] or with primers rD1 and fD1 as described by Herrera-Cervera et al. [54]. *rec*A and *atp*D gene fragments were amplified following Gaunt et al.’s methods [55]. For *rec*A gene amplification, recA6, recA63, recA504, and recA555 primers were used; for *atp*D gene amplification, atpD273, atpD294 and atpD771 primers were used. *gln*II gene was amplified using glnR and glnF primers [56]. *nod*C and *nif*H genes were amplified following Laguerre et al.’s methods [57], using primers nodCFn, nodCF2, nodCF4, and nodCFu, nodCI, nifHF, and nifHR primers, respectively. Newly designed primers (Table 1) were used when amplification with the previous primers was not possible. For these PCR reactions primers were used at a concentration of 5 μM, together with 15.8 μL of Qiagen kit Taq mix solution (2.5 U of taq polymerase, 1.5 mM of MgCl_2_, 200 μM of the different dNTP), and approximately 20 ng of genomic DNA, in a final volume of 20 μL. Amplifications were performed using Eppendorf Mastercyler Gradient thermocycler. For *rec*A amplification, samples were incubated for 5 min at 95 °C followed by 35 rounds of thermocycling conditions (95 °C for 45 s, 59 °C for 1 min, and 72 °C for 1 min 30 s) and a final extension phase (72 °C, 7 min). For *atp*D, samples were incubated for 5 min at 95 °C followed by 35 rounds of thermocycling conditions (95 °C for 45 s, 53 °C for 1 min and 72 °C for 2 min, using atpD183/atpD872 primers, or 95 °C for 45 s, 58 °C for 1 min, and 72 °C for 2 min, using atpD133/atpD762 primers) and a final extension phase (72 °C for 7 min). Primer 16SIR was only used for fully sequencing the 16S amplicon from some strains. The amplified DNA fragments were electrophoresed and extracted from agarose with the QIAEXII kit (Qiagen; Venlo, Netherlands) and sequenced with an ABI373 automated sequencer in the sequencing service of the “Instituto de Parasitología y Biomedicina, Lopez Neyra” CSIC, Spain. Alternatively, PCR products were sequenced using an ABI 3730 XL automated sequencer, by Stabvida, Caparica, Portugal.

### 2.4. NCBI Accession Numbers

Sequences obtained previously were deposited in the NCBI database. Below a list of all accession codes is presented next to the correspondent strain, for each gene.

- 16S rRNA
  LpA5a: MK611706; LpA5b: MK611741; LpA6: MK611742; LpA7b: MK611744; LpA8: MK611745; LpA9: MK611746; LpA10: MK611747; LpA11: MK611748; LpA12: MK611749; LpA13a: MK611750; LpB5b: MT071928; LpB5d: MT071934; LpB9a: MT071930; LpB10a: MT071937; LpB10b: MT071935; LpB10c: MT071938; LpB10d: MT071936; LpB12a: MT071929; LpB12b: MT071924; LpB13: MT071932; LpB14a: MT071927; LpB14b: MT071931; LpB15b: MT071926; LpB16c: MT071939; LpB16d: MT071925; LpB17b: MT071933; LpB19a: MK611753; LpB23b: MK611754; LpB26: MK611755.
- RecA
  LpA5a: MK671539; LpA5b: MK690337; LpA6: MK671540; LpA7b: MK671541; LpA8: MK690338; LpA9: MK671542; LpA11: MK671543; LpA13a: MK690339; LpB19a: MK671547; LpB23b: MK671548; LpB26: MK671549.
- AtpD
  LpA5a: MK671550; LpA5b: MK690340; LpA6: MK671551; LpA7b: MK671553; LpA8: MK690341; LpA9: MK671554; LpA10: MK690342; LpA11: MK671555; LpA13a: MK671557; LpB19a: MK671560; LpB23b: MK671561; LpB26: MK671562.
- GlnII
  LpA8: MK690343; LpA9: MK690344; LpA11: MK671563; LpA13b: MK690346; LpA14: MK690347; LpB19a: MK671564; LpB23b: MK671565; LpB26: MK671566.
- NodC
  LpA11: MK671567; LpB19a: MK671568; LpB23b: MK671569; LpB26: MK671570.
- NifH
  LpA11: MK690348; LpB19a: MK671571; LpB23b: MK671572; LpB26: MK671573.

### 2.5. Phylogenetic Analysis

Nucleotide sequences were visualized in a chromatogram and edited manually, as necessary, with Chromas lite program (version 2.1.1). Homologous sequences in NCBI Genebank database were identified using BLASTn tool. Alignments were performed using ClustalW and phylogenetic reconstruction was performed using IQ-tree [58] on XSEDE [59] and CIPRES platforms [60], with the Maximum Likelihood (ML), ModelFinder [61], and Ultra-Fast bootstrap [62] methods. An appropriate outgroup was selected to perform the ML method. The bootstrap confidence values were based on 1000 replications. Tree visualization was performed using the Interactive Tree Of Life tool [63].

### 2.6. Nodulation Tests

The nodulation ability of isolates was tested in the original host *L. parviflorus* (seeds collected from location B), as well as in related legumes: *L. uliginosus* cv. Sunrise; *L. tenuis* cv. Pampa INTA; *L. tenuis* cv. Esmeralda; *L. corniculatus* cv. San Gabriel; *Lupinus luteus* cv. Nacional; and *Glycine max* 104904B-Próvida. Seeds were surface sterilized according to Hahn and Somasegaran and Hoben [48,49]. After 1–2 h in sterilized water, seeds were transferred to 0.8% *w v*^−1^ agar-water plates for 1–2 (*Lotus* seeds) or 2–4 days (*Lupinus and Glycine* seeds). After germination, seedlings were transferred to slants (*Lotus* spp.) or to Leonard-type jars (*Lupinus* and *Glycine*) containing N-free Jensen plant nutrient medium [64]. Each isolate was inoculated by applying a 1 mL bacterial suspension (approximately 10^9^ bacterial cells in ¼ Jensen medium), on the roots of each seedling (3 replicates per each bacteria–host combination). Additionally, controls (TN) fertilized with 1 mL (*Lotus* spp.) or 2 mL (*Lupinus* and *Glycine*) of 4.45 mM KNO_3_^−^ (final concentration), and non-inoculated (T0) supplemented with 1 mL of N-free liquid Jensen medium (¼ diluted), were also prepared. Plants were grown in environmental chambers under controlled conditions (16/8 h photoperiod and 23/18 °C). *Lupinus* and *Glycine* plants were transferred to the greenhouse after 4 weeks of growth in the environmental chambers. After 8 weeks of growth, the ability to nodulate and fix nitrogen was evaluated by visual inspection of the nodules, and by comparison of plant sizes and aspect with T0 and TN controls. In addition, dry weights of aerial biomass were measured, after drying at 80 °C for 2 days, to determine the relative symbiotic effectiveness, as described by Ferreira and Marques [65].

### 2.7. Tests for Plant-Growth-Promotion (PGP) Traits

Qualitative tests for classical PGP activities, such as phosphate (P) solubilization, siderophore production, antagonism against phytopathogens, and production of lytic enzymes (pectinases and celullases) were carried out. Solubilization of tricalcium phosphate was tested in YED-agar plates supplemented with 0.2% of Ca_3_PO_4_ [66]. Isolates with halos around the colonies were considered as P solubilizers. The presence of siderophores was tested in TY media [67] using a CAS solution, which contains iron conjugated with a dye as a color indicator, as described by Pérez-Miranda et al. [68]. Isolates that formed orange halos were considered siderophore producers. For the antagonistic activity against phytopathogens, a portion of *Phytophthora cinnamomi* or *Botryosphaeria cortícola* mycelium was inoculated in the center of YMA plates. After 2 days of incubation at 27 °C in the dark, the bacterial isolates were inoculated in the periphery of the plate and incubated for 10 days at 27 °C in the dark. The antagonistic activity was considered when inhibition of the phytopathogens growth was visualized. Production of cellulases or pectinases were tested in TY media supplemented with 0.2% *w v*^−1^ of carboxymethylcellulose (CMC) or 0.5% *w v*^−1^ of Pectin, following procedures by Verma, Ladha, and Tripathi [69]. The appearance of halos around the colonies was accounted for the presence of the respective lytic enzymes (cellulases or pectinases).

## 3. Results

### 3.1. Bacterial Diversity

A total of 13 isolates from site A (named LpA) and 27 from site B (named LpB) were obtained from the root nodules of *L. parviflorus* plants. In some instances, more than one colony type (colony morphology, distinct congo red absorption, etc.) coming from a single nodule were visualized, (named types a, b, c, etc.; e.g., LpA5a and LpA5b). A broad range of congo red staining levels and growing velocity were observed among the isolated colonies (Table 2).

The majority of the isolates were fast or very fast growers and did not absorb the congo red stain. The only isolates that were slow growers and did not absorb congo red were LpA11 from site A and LpB19a, LpB23b, and LpB26 from site B. The appearance of visible colonies within 24 h that also absorbed congo red (Table 2) was an indication that some bacteria might not be rhizobia [49].

REP fingerprints were obtained for all isolates except for four (LpB5c, LpB5e, LpB9b, and LpB23a). Fingerprints were composed of 2–18 bands with sizes between 200–8.000 bp (Figure S1). Considering a similarity index of 80% for strain differentiation [16], the analysis revealed 29 different clusters among the 36 isolates tested. In several instances, isolates obtained from the same nodule that had been considered visually different still displayed identical fingerprints (e.g., LpA7a and LpA7b; LpB5b, LpB5g, and LpB5f; LpB16a, LpB16b, and LpB16d). In other instances, identical fingerprints were shown by isolates originating from different nodules (LpA12, LpA13b, and LpA14; Figure S1).

### 3.2. Phylogenetic Analyses

#### 3.2.1. 16S rRNA Gene

A single 1500 bp fragment, corresponding to almost full-length 16S rRNA gene, was PCR amplified of one isolate from each cluster (29 in Total; Figure S1) and further sequenced.

**Figure 2:**
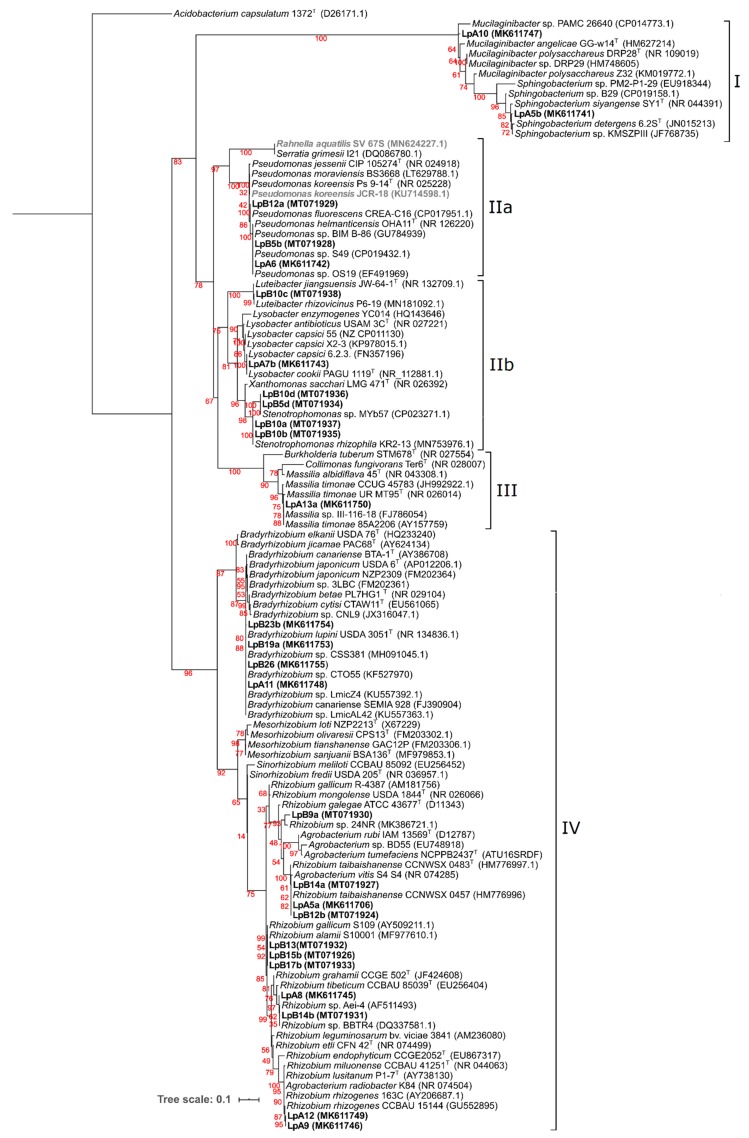
Phylogeny of 16S rRNA based on 920 positions. Analyses were conducted with IQ-tree using Maximum Likelihood (ML) and ModelFinder (Best model: TIM3+F+I+G4) methods. Confidence levels of bootstrap are presented in red near each node. NCBI Genebank accession codes are presented next to each strain. *Acidobacterium capsulatum* 1372^T^ (D26171.1), a bacterium from the Acidobacteria phylum, was selected as an outgroup. Isolates obtained from *L. parviflorus* nodules are in bold.

Aligned sequences of 920 nt positions were used to construct the phylogenetic ML tree shown in Figure 2, where four major clades can be observed. Isolates LpB16c and LpB16d were not included in this tree because only small sequence stretches from the 16S rRNA gene could be obtained. Nonetheless, highest sequence identities obtained were still included in the tree and are represented in bold and grey.

Clade I included bacteria belonging to the Bacteroidetes phylum (*Sphingobacteriaceae*) family. Isolates LpA5b and LpA10 were placed in this clade, and their closest relatives were *Sphingobacterium* sp. KMSZPIII and *Mucilaginibacter* sp. PAMC 26640, respectively. Clade II contained bacteria from the gamma-proteobacteria class. *Pseudomonadaceae* and *Enterobacteriaceae* families comprised subclade IIa, where LpA6, LpB5b, and LpB12a were included. Isolates LpA6 and LpB5b were grouped with *Pseudomonas helmanticensis* OHA11 and other unclassified *Pseudomonas* spp. The closest relative of LpB12a was *Pseudomonas fluorescens* CREA-C16. LpB16d and LpB16c (not included in this tree) would have the highest identity with *Pseudomonas koreensis* JCR-18 (100% identity) and *Rahnella aquatilis* SV_67S (100% identity), respectively. Subclade IIb was composed by *Rhodanobacteraceae* and *Xanthomonadaceae* families. Isolate LpB10c was grouped with *Luteibacter rhizovicinus* P6-19. Isolates LpB5d and LpB10d were clustered together and with *Stenotrophomonas* sp. MYb57. Isolates LpB10a and LpB10b were clustered together and with *Stenotrophomonas rhizophila* KR2-13. Isolate LpA7b was clustered with *Lysobacter cookii* PAGU 1119 and *L. capsici* 6.2.3. Clade III included bacteria from the beta-proteobacteria class (*Oxalobacteraceae* family). LpA13a was the only isolate placed in this clade and was grouped with *Massilia timonae* 85A2206 and *Massilia* sp. II-116-18. Clade IV comprised of bacteria belonging to the alpha-proteobacteria class (*Bradyrhizobiaceae Rhizobiaceae* and *Phyllobacteriaceae* families). Isolates LpA11, LpB19a, LpB23b, and LpB26 were clustered with *Bradyrhizobium canariense* SEMIA 928, *Bradyrhizobium lupini* USDA 3051, and other *Bradyrhizobium* strains isolated from genistoid legumes. Isolate LpB9a was clustered with *Rhizobium* sp. 24NR. Isolates LpA5a, LpB14a, and LpB12b were clustered in a group with *Rhizobium taibaishanense* CCNWSX 0457. LpB13, LpB15b, and LpB17b were clustered together and with *Rhizobium gallicum* S109 and *Rhizobium alamii* S10001. Isolate LpB14b was grouped with *Rhizobium* sp. BBTR4, while LpA8 was grouped isolated in a sister cluster. Isolates LpA9 and LpA12 were clustered in a separated group close to *Rhizobium rhizogenes* CCBAU 15144

#### 3.2.2. Protein-Encoding Housekeeping Genes

Since 16S rRNA gene cannot provide the best phylogenetic resolution for some bacteria, like in the case of Bradyrhizobia, MLSA of several protein-encoding housekeeping genes was performed. Only site B strains that nodulated (LpB19a, LpB23b, and LpB26; Table 3) were considered for further phylogenetic analysis, while all distinctive isolates by REP-PCR (80% similarity) and 16S rRNA gene sequence from site A (LpA5a, LpA5b, LpA6, LpA7b, LpA8, LpA9, LpA10, LpA11, and LpA13a) were considered. From these, only LpA8, LpA9, LpA11, LpB19a, LpB23b, and LpB26 genomic DNAs produced amplicons for all housekeeping genes tested, with sizes of approximately 500 bp for *rec*A and *atp*D, and 700bp for *gln*II. The rest of the isolates failed mainly to produce amplicons for *gln*II gene, which unlike *gln*A is not ubiquitous in bacteria [70]. For isolates LpA5b, LpA6, LpA10, and LpA13a we could obtain an *atp*D amplicon of approximately 700 bp only after using the newly designed primers (atpD183 and atpD872 for LpA5b and LpA10, and atpD133 and atpD762 for LpA6 and LpA13a; see Table 1). Also, the newly designed *rec*A primers allowed us to obtain a *rec*A amplicon of almost 600 bp from isolate LpA5b.

**Figure 3:**
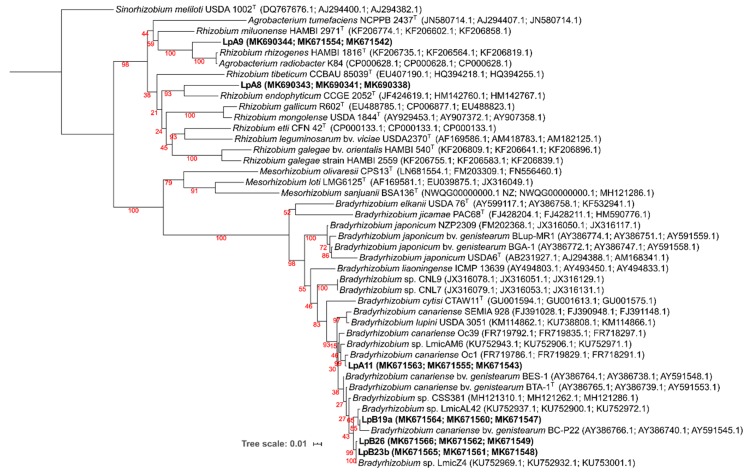
Phylogeny of concatenated sequences of *gln*II (300 nt), *atp*D (328 nt), and *rec*A (333 nt) genes, based on a total of 968 nt. Bootstrap values are shown in red at each node. Analyses were conducted with IQ-tree using Maximum Likelihood (ML) and ModelFinder (Best model: TIM+F+I+G4) methods. NCBI Genebank accession codes are shown. *Sinorhizobium meliloti* USDA 1002^T^ (DQ767676.1; AJ294400.1; AJ294382.1) was selected as an outgroup. Isolates obtained from *L. parviflorus* nodules are in bold.

The protein encoding housekeeping genes phylogeny was congruent with the 16S rRNA phylogeny at the genus and also very often at the species level. As there are few sequences available in the NCBI Genebank database for genera *Sphingobacterium, Lysobacter*, *Mucilaginibacte*r, and *Massilia,* most of the sequences for these genera were gathered from genome sequencing projects. For the isolates from which all three housekeeping genes could be amplified, a ML phylogenetic tree was generated using concatenated sequences of *gln*II, *atp*D, and *rec*A genes (Figure 3). The clustering of isolates LpA11, LpB19a, LpB23b, and LpB26 within *Bradyrhizobium* was confirmed to be closely related to *B. canariense*. The closest relative of LpA11 was *B. canariense* Oc1 (99.6% sequence identity), a strain isolated from *Ornithopus compressus* in Sardinia, Italy [71]. LpB19a phylogenetic position was less resolved. It was grouped in a low confidence cluster with *Bradyrhizobium canariense* BC-P22 (98.3% identity), a strain isolated from *Chamaecytisus proliferus* in the Canary Islands [72], while sharing a higher identity with *Bradyrhizobium* sp. LmicAL42 (99.6% identity), a strain isolated from *Lupinus micranthus* nodules in Spain [73]. LpB23b and LpB26 were clustered together in a highly supported cluster with *Bradyrhizobium* sp. LmicZ4 (100% similarity for LpB23b and 99.9% of similarity for LpB26), a strain isolated from *Lupinus micranthus* nodules in Algeria [73]. On the other hand, isolates LpA8 and LpA9 were again clustered within the *Rhizobium/Agrobacterium* group, albeit not matching any previously described species.

For those isolates that were negative for *gln*II amplification, ML phylogenetic trees of *rec*A and *atp*D genes were constructed (Figure S2). Isolate LpA5a was clustered with *Rhizobium taibaishanense* HAMBI 3414T (now *Allorhizobium taibaishanense*, in accordance with Bourebaba et al. [74]), having a similarity of 98.2% in *rec*A and 98.6% *atp*D gene, respectively. LpA5b was placed in a clade of *Sphingobacterium* spp., having *Sphingobacterium* sp. B29 as the closest relative (98.5% identity in the *atp*D gene and 92.1% identity in the *rec*A gene). LpA6 was clustered with *Pseudomonas* spp., having *Pseudomonas koriensis* DSM 11610^T^ (99.7% identity) and *Pseudomonas jessenii* DSM 17150^T^ (96.1% identity) as the closest relatives in the *atp*D and *rec*A gene trees, respectively. Isolate LpA7b was placed in the *Lysobacter* genus, sharing the highest identities with *L. capsici* 55 (99.2% in the *atp*D gene and 99.3% in *rec*A gene). Isolate LpA10 was clustered in a single branch within the *Mucilaginibacter* clade, sharing only 85.4% and 84.8% with *Mucilaginibacter mallensis* MP1X4 and *Mucilaginibacter* sp. PAMC 26640, respectively, in the *atp*D gene. Lp13a was grouped with *Massilia* spp., sharing 100% of similarity, in both genes, with *M. timonae* CCUG 45783.

#### 3.2.3. Symbiotic Genes: *nod*C and *nif*H

**Figure 4:**
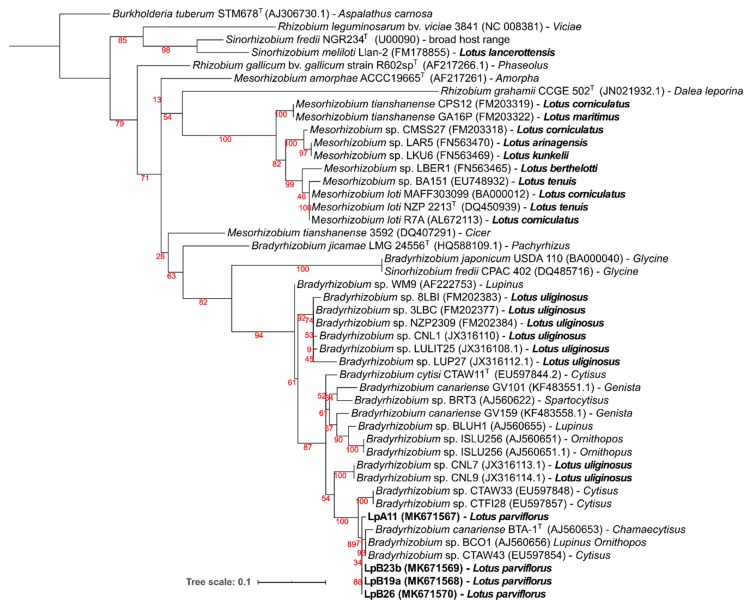
Phylogenetic tree of *nod*C gene sequences. Analyses were conducted with IQ-tree using Maximum Likelihood (ML) and ModelFinder (Best model: HKY+F+G4) methods), for a total of 203 positions. Bootstrap values are shown in red at each node. NCBI Genebank accession codes and original legume host of isolation are indicated. *Burkholderia tuberum* STM678^T^ (AJ306730.1), a bacterium from the beta-proteobacteria class that nodulates *Aspalathus carnosa*, was selected as an outgroup. *Lotus* spp. and isolates obtained from *L. parviflorus* nodules are in bold.

**Figure 5:**
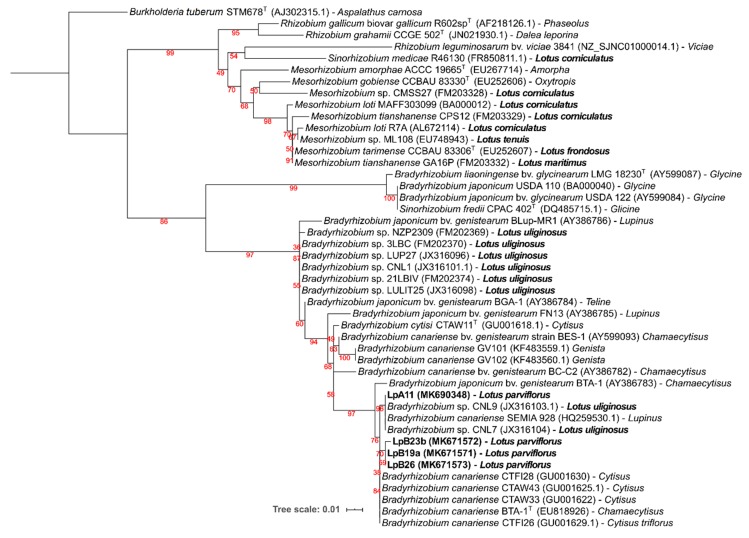
Phylogenetic tree of *nifH* gene sequences. Analyses were conducted with IQ-tree using Maximum Likelihood (ML) and ModelFinder (Best model: K2P+G4) methods), for a total of 203 positions. Bootstrap values are shown at each node. NCBI Genebank accession codes and original legume host of isolation are indicated. *Burkholderia tuberum* STM678^T^ (AJ302315.1), a bacterium from the beta-proteobacteria class that nodulates *Aspalathus carnosa*, was selected as an outgroup. *Lotus* spp. and isolates obtained from *L. parviflorus* nodules are in bold.

Symbiotic *nodC* and *nifH* gene amplicons were obtained only from strains LpA11, LpB19a, LpB23b, and LpB26, which matched their nodulation ability (Table 3). For both gene phylogenies (Figure 4 and Figure 5), these isolates were clustered in a well-supported clade, comprising of *B. canariense* and other *Bradyrhizobiu*m sp. strains isolated from genistoid legumes. The closest *Lotus* rhizobia were isolates from *L. uliginosus*, which is in line with the results of the nodulation tests (Table 3).

### 3.3. Nodulation Tests

Symbiotic ability (nodulation and effectiveness) was tested for all isolates in various *Lotus* species. Isolates that nodulated *Lotus* plants were additionally tested in *Lupinus luteus* and *Glycine max* plants (Table 3). Only isolates LpA11 from sampling site A, and LpB19a, LpB23b, and LpB26 from site B were able to nodulate any of the *Lotus* spp. tested. LpA11 could nodulate and fix nitrogen in *L. uliginosus*, *L. parviflorus,* and *L. luteus*. It also formed non-fixing nodules in *L. corniculatus* and *L. tenuis* cv. Esmeralda, as judged from nodule appearance (white color) and plants dry weight. No nodulation was observed in *L. tenuis* cv. INTA PAMPA, and *G. max*. LpB19a, LpB23b, and LpB26 isolates displayed a Nod^+^ Fix^+^ phenotype in *L. uliginosus*, *L. parviflorus,* and *L. luteus,* as well as a Nod^+^Fix^−^ phenotype in *L. corniculatus* and *L. tenuis* cv. INTA PAMPA. Similar to LpA11, none of them formed nodules in *G. max*. The results of the symbiotic tests are in accordance with the symbiotic genes phylogenies, that placed the *L. parviflorus* symbionts close to rhizobia from genistoid legumes and *L. uliginosus*, but distant from other *Lotus* spp. and *Glycine* rhizobia (see Figure 4 and Figure 5).

### 3.4. PGP Traits

Nearly 40% of the non-nodulating isolates displayed one or more activities related with plant growth promotion or protection (Table 4; Figures S3 and S4). Particularly abundant was the production of cellulases, often by strains that also showed antagonistic activity against fungal phytopathogens (isolates LpA7a, LpA7b, LpB16a, LpB16b, LpB16d, and LpB23a). In contrast, only one isolate (LpB16d) showed significant P solubilization capacity, whereas 4 isolates (LpA6, LpB12a, LpB5b, and LpB5c) showed siderophore production under our experimental conditions (Table 4). These results, together with the phylogenetic analyses described above, indicate that the non-nodulating bacteria isolated from *L. parviflorus* nodules are common soil and rhizosphere inhabitants, which can eventually exhibit plant beneficial traits. For instance, isolates LpA7a and LpB16d, which showed antagonistic activity against phytopathogens and lytic enzyme production (Table 4), are closely related to *Lysobacter capsici* and *Pseudomonas koreensis*, respectively. These species are well known for a suppressive activity on several plant pathogens [46,75,76,77,78]. Moreover, isolates LpA6, LpB5b, LpB12a, and LpB16d, which showed siderophore production, are closely related to *Pseudomonas* spp. (*Pseudomonas koreensis* and *Pseudomonas fluorescens*) that are commonly reported to show this trait [77,79,80,81]. Similarly, phosphate solubilization was shown by the pseudomonad isolate LpB16d that is related to *P. koreensis*, which has also been shown previously to solubilize phosphate [82,83,84].

## 4. Discussion

We found a great diversity of bacteria inside nodules of wild *L. parviflorus* plants growing in two different locations in Portugal. Intriguingly, only four of these isolates (10%) were able to nodulate the original host *L. parviflorus*. These four isolates are slow growers, and phylogenetically related to *B. canariense* and other *Bradyrhizobium* strains isolated from legumes, such as *L. uliginosus*, *Ornithopus* and other genistoid legumes (e.g., *Lupinus*) root nodules. Indeed, *L. parviflorus* bradyrhizobia were also able to effectively nodulate *L. uliginosus* and *L. luteus*. The other 30 isolates were not able to nodulate any of the legume hosts tested. Often, the symbiotic bradyrhizobia and the non-nodulating bacteria were isolated from the same nodule, suggesting that the later could be opportunistic nodule inhabitants. Almost all these non-nodulating bacteria displayed faster growth rates than the nodulating bradyhrizobia, which would explain why they were much more frequently isolated than the actual symbiotic bradyrhizobia. However, we have isolated slow-growing bradyrhizobia from other related legumes, such as *L. uliginosus* [9,16], following similar procedures, without observing such an abundance of fast-growing bacteria inside the nodules. Thus, for some unknown reason the nodules of *L. parviflorus* seem more frequently invaded by non-nodulating opportunistic bacteria than other *Lotus* spp. Alternatively, other legume nodule endophytes may be unculturable or unable to grow in the YMA medium generally used for rhizobial isolation. Nevertheless, the high abundance of fast-growing nodule endophytes has seriously hindered our goal of describing the genetic diversity among *L. parviflorus* rhizobia. The presence of these fast-growing strains when cultivated could inhibit the growth of the bradyrhizobia that are able to nodulate *L. parviflorus*. Fast growers are able to spread on the agar growth media, likely disturbing or even inhibiting the growth of bradyrhizobia. In addition, some of the isolated strains could display active motility in solid surfaces (swarming, gliding, sliding), e.g., *Lysobacter* spp. possess gliding motility [46], that can further facilitate their spreading on the agar surface. For future studies, approaches to favor either the selective isolation of the bradyrhizobia or the counterselection of other nodule bacteria, should be implemented.

Besides nitrogen fixation, some authors also show other PGP activities from rhizobia, such as phosphate solubilization, siderophore production, and biocontrol activity [40,85,86,87]. However, we did not find any PGP activity (besides nitrogen fixation) from the nodulating bradyrhizobia, and only one non-nodulating *Rhizobium* spp. strain (LpB12b) showed lytic enzyme production. Indeed, the use of co-inoculations of rhizobia and other bacteria more specialized for other PGP traits is a common practice in biotechnological applications in agriculture [88,89,90,91]. All *L. parviflorus* nodule bacteria were related to previously described soil or plant-associated taxa. We observed that many of these nodule inhabitants could exhibit activities potentially beneficial for plant growth and health, including phosphate solubilization, production of siderophores and lytic enzymes, as well as antagonism against two well-known phytopathogens, *Phytophthora cinnamomi* and *Botryosphaeria cortícola.* Endophytic bacteria with these beneficial activities are frequently found in legume tissue, including nodules [92,93,94]. The isolation of bacteria other than rhizobia from the interior of the legume nodules has been increasingly reported in precious years [28,30,31,32,33,95]. Some plant species seem more prone to harbor a high amounts and diversity of non-rhizobia inside their nodules, as reported by De Meyer et al. [28], who studied 654 isolates from the nodules of 30 different plant species. Moreover, there are certain endophytic bacteria that have been frequently isolated from legume root nodules, e.g., *Micromonospora sp.* [33], *Bacillus* sp., and *Pseudomonas* sp. [28]. Remarkably, [34] have shed some light in the understanding of this phenomenon. By following the progression of *L. japonicus* nodule infection and colonization by *M. japonicum* R7A and endophytic strains, these authors found that the endophyte’s ability to infect and colonize nodules was discriminated and controlled by the plant. Exopolysaccharides produced by endophytic bacteria and early symbiotic plant genes play a vital role to enable and favor their nodule invasion and colonization through infection threats initiated by *M. japonicum* R7A. This determinant molecular crosstalk between endophytes and plants could explain why some legume plants are more prone to be colonized by certain endophytes. The fact that we isolated closely related bacteria from two different locations separated by nearly 200 Km (see Figure 2, isolates LpA5a, LpB12b, and LpB14a; LpA6 and LpB5b; LpA8 and LpB14b) argues for a potential selectivity by the host plant. In our hands, *L. parviflorus* nodules seem more predisposed to be colonized by non-rhizobial bacteria than other related species such as *L. uliginosus* [9,15,28]. Likewise, *L. corniculatus* has also been reported to have nodules frequently colonized by endophytic bacteria [17,96,97], albeit not to the extent we have found for *L. parviflorus* in this study.

It would be interesting to know if any of *L. parviflorus* nodule bacteria can also be found inside other plant organs, and whether there is some type of selectivity or favoritism towards certain taxa. In contrast with legume–rhizobia symbiosis, interactions of legumes with other endophytes are just starting to be unraveled. Our results reinforce the notion that legume root nodules can host complex bacterial communities that could express nitrogen fixation and additional plant growth promoting activities.

## Figures and Tables

**Table 1:** Newly designed primers for PCR and sequencing.

Primer	Gene	Position *	Direction	Length	Sequence (5′–3′)
recA107	*rec*A	107	Forward	23 bp	TTAGGTGATACTGCCRTBGARCC
recA593	*rec*A	593	Reverse	24 bp	GGGTTWCCGAACATTACRCCRATT
atpD183	*atp*D	183	Forward	24 bp	CGTGTTCGTRCHATTGCBATGGAY
atpD872	*atp*D	872	Reverse	20 bp	GGCATACGGCCCAGCAGTGC
atpD133	*atp*D	133	Forward	23 bp	GACCCTGGAAGTTCAGCAGCAGC
atpD762	*atp*D	762	Reverse	20 bp	GGCATACGGCCCAGCAGTGC
16SIR	16SrRNA	875	Reverse	21 bp	AAACACATGCTCCACCGCTTG

* Position relative to the start of *rec*A gene of *Sphingobacterium spiritivorum* ATCC 33861 (ACHA02000002.1; *rec*A primers); relative to the start of *atp*D gene of *Sphingobacterium spiritivorum* ATCC 33861 (ACHA02000004.1; atpD183 and atpD872), or *Pseudomonas fluorescens* Pf0-1 (CP000094.2; atpD133 and atpD762). Position relative to the start of 16S rRNA gene of *Rhizobium leguminosarum* bv. *trifolii* WSM1689 (NZ_CP007045.1).

**Table 2:** Congo red absorption and growth speed of bacteria isolated from *L. parviflorus* nodules.

	Isolates	Congo Red Absorption	Growing Velocity
Sample Spot A	LpA5a, LpA6, LpA8, LpA9, LpA12, LpA13b, LpA14, LpA5b	−	Fast
	LpA7a, LpA7b, LpA10, LpA13a	+	Moderate
	LpA11	−	Slow
Sample Spot B	LpB5b, LpB5c, LpB5e, LpB5f, LpB5g, LpB12a, LpB16a, LpB16b, LpB16c, LpB16d	−	Very fast
	LpB10d	+	
	LpB9a, LpB9b, LpB12b, LpB13, LpB14a, LpB14b, LpB15b¸ LpB17b	−	Fast
	LpB5d, LpB10a LpB10b	+	
	LpB19a, LpB23b, LpB26	−	Slow
	LpB10c, LpB23a	+	

Very fast: colonies visible within 24 h; Fast: colonies visible within 1–2 days; Moderate: colonies visible within 3–4 days; Slow: colonies visible within 5–8 days.

**Table 3:** Host range assays performed in different legume host plants inoculated with the different bacterial isolates.

Isolates	Symbiotic Phenotype						
	*Lotus* Group I			*Lotus* Group II		*Lupinus*	*Glycine*
	Lc	Lt P	Lt E	Lu	Lp	Ll	Gm
LpA11	Fix^−^	Nod^−^	Fix^−^	Fix^+^	Fix^+^	Fix^+^	Nod-
LpB19a, LpB23b, LpB26	Fix^−^	Fix^−^	NT	Fix^+^	Fix^+^	Fix^+^	Nod-
LpA5a, LpA5b, LpA6, LpA7a, LpA7b, LpA8, LpA9, LpA10, LpA12, LpA13a, LpA13b, LpA14	Nod^−^	Nod^−^	Nod^−^	Nod^−^	Nod^−^	NT	NT
LpB5b, LpB5c, LpB5d, LpB5e, LpB5f, LpB5g, LpB9a, LpB9b, LpB10a, LpB10b, LpB10c, LpB10d, LpB12a, LpB12b, LpB13, LpB14a, LpB14b, LpB15b, LpB16a, LpB16b, LpB16c, LpB16d, LpB17b, LpB23a	Nod^−^	Nod^−^	NT	Nod^−^	Nod^−^	NT	NT

Lc: *L. corniculatus* cv. San Gabriel; LtP*: Lotus tenuis* cv. Inta PAMPA; Lt E: *Lotus tenuis* cv. Esmeralda; Lu: *L. uliginosus* cv. Sunrise; Lp: *L. parviflorus*; Ll: *Lupinus luteus*; Gm: *Glycine max*. NT: Not tested; Nod^−^: lack of nodulation; Fix^−^: Nodulation occurred, but no nitrogen fixation; Fix^+^: Partially efficient nitrogen fixation; Fix^+^: Efficient nitrogen fixation.

**Table 4:** Plant growth promotion (PGP) traits: Phosphate solubilization, siderophore production, antagonistic activity against phytophatogens, and lytic enzymes production. Only isolates that showed at least one activity assayed are presented, even though all isolates were tested.

Isolates	P sol	Sid Production		Antagonistic Activity		Lytic Enzymes Production	
				*P. cinammomi*	*B. corticola*	Cel	Pect
LpB16d	+		−	+	+	+	−
LpA7a, LpA7b, LpB16a, LpB16b	−		−	+	+	+	−
LpB23a	−		−	−	+	+	+
LpB9b	−		−	−	−	+	+
LpB5b,	−		+	−	−	+	−
LpB12b, LpB10b, LpB5f	−		−	−	−	+	−
LpA6, LpB12a, LpB5c	−		+	−	−	−	−
LpB5e	−		−	−	−	−	−

P sol: Phosphate solubilization; Sid production: Siderophore production; cel: Cellulase activity; Pect: Pectinase activity.

## Supplementary Materials

The following are available online at https://www.mdpi.com/2076-2607/8/4/499/s1, Figure S1: UPGMA dendrogram, Figure S2: Phylogeny of *atp*D and *rec*A genes, Figure S3: Inhibition of *P. cinnamomi* and *B. corticola*, Figure S4: In vitro plant growth promoting rhizobacteria (PGPR) activities.

## References

[1] Allan G.J., Francisco-Ortega J., Santos-Guerra A., Boerner E., Zimmer E.A. (2004). Molecular phylogenetic evidence for the geographic origin and classification of Canary Island Lotus (Fabaceae: Loteae). Mol. Phylogenetics Evol..

[2] Díaz P., Borsani O., Monza J. (2005). *Lotus*-related species and their agronomic importance. Lotus japonicus Handbook.

[3] Márquez A. (2005). Lotus Japonicus, A General Introduction.

[4] Sato S., Nakamura Y., Kaneko T., Asamizu E., Kato T., Nakao M., Sasamoto S., Watanabe A., Ono A., Kawashima K. (2008). Genome structure of the legume, *Lotus japonicus*. DNA Res..

[5] Monza J., Fabiano E., Arias A. (1992). Characterization of an indigenous population of rhizobia *nodulating Lotus corniculatus*. Soil Biol. Biochem..

[6] Pankhurst C.E., Craig A.S., Jones W.T. (1979). Effectiveness of Lotus Root Nodules: I. morphology and flavolan content of nodules formed on *Lotus pedunculatus* by fast-growing *Lotus* rhizobia. J. Exp. Bot..

[7] Lorite M.J., Muñoz S., Olivares J., Soto M.J., Sanjuán J. (2010). Characterization of strains unlike *Mesorhizobium loti* that nodulate lotus spp. in saline soils of Granada, Spain. Appl. Environ. Microbiol..

[8] Irisarri P., Milnitsky F., Monza J., Bedmar E.J. (1996). Characterization of rhizobia nodulating Lotus subbiflorus from Uruguayan soils. Plant Soil.

[9] Lorite M.J., Videira e Castro I., Muñoz S., Sanjuán J. (2012). Phylogenetic relationship of *Lotus uliginosus* symbionts with bradyrhizobia nodulating genistoid legumes. FEMS Microbiol. Ecol..

[10] Cooper J.E., Wood M., Bjourson A.J. (1985). Nodulation of *Lotus pedunculatus* in acid rooting solution by fast- and slow-growing rhizobia. Soil Biol. Biochem..

[11] Estrella M.J., Muñoz S., Soto M.J., Ruiz O., Sanjuán J. (2009). Genetic diversity and host range of rhizobia nodulating Lotus tenuis in typical soils of the Salado River Basin (Argentina). Appl. Environ. Microbiol..

[12] Lorite M.J., Donate-Correa J., del Arco-Aguilar M., Pérez Galdona R., Sanjuán J., León-Barrios M. (2010). *Lotus* endemic to the Canary Islands are nodulated by diverse and novel rhizobial species and symbiotypes. Syst. Appl. Microbiol..

[13] León-Barrios M., Lorite M.J., Donate-Correa J., Sanjuán J. (2009). *Ensifer meliloti* bv. *lancerottense* establishes nitrogen-fixing symbiosis with *Lotus* endemic to the Canary Islands and shows distinctive symbiotic genotypes and host range. Syst. Appl. Microbiol..

[14] Merabet C., Martens M., Mahdhi M., Zakhia F., Sy A., Le Roux C., Domergue O., Coopman R., Bekki A., Mars M. (2010). Multilocus sequence analysis of root nodule isolates from *Lotus arabicus* (Senegal*), Lotus creticus*, *Argyrolobium uniflorum* and *Medicago sativa* (Tunisia) and description of *Ensifer numidicus* sp. nov. and *Ensifer garamanticus* sp. nov. Int. J. Syst. Evol. Microbiol..

[15] Lorite M.J., Estrella M.J., Escaray F.J., Sannazzaro A., Videira e Castro I.M., Monza J., Sanjuán J., León-Barrios M. (2018). The Rhizobia-*Lotus* Symbioses: Deeply Specific and Widely Diverse. Front. Microbiol..

[16] Batista L., Tomasco I., Lorite M.J., Sanjuán J., Monza J. (2013). Diversity and phylogeny of rhizobial strains isolated from *Lotus uliginosus* grown in Uruguayan soils. Appl. Soil Ecol..

[17] De Meyer S.E., Van Hoorde K., Vekeman B., Braeckman T., Willems A. (2011). Genetic diversity of rhizobia associated with indigenous legumes in different regions of Flanders (Belgium). Soil Biol. Biochem..

[18] Avontuur J., Palmer M., Beukes C.Y., Chan W., Coetzee M., Blom J., Stępkowski T., Kyrpides N.C., Woyke T., Shapiro N. (2019). Genome-informed *Bradyrhizobium* taxonomy: Where to from here?. Syst. Appl. Microbiol..

[19] Ormeño-Orrillo E., Martínez-Romero E. (2019). A Genomotaxonomy View of the *Bradyrhizobium* Genus. Front. Microbiol..

[20] Bradyrhizobium. http://www.bacterio.net/bradyrhizobium.html.

[21] Evans W.R., Fleischman D.E., Calvert H.E., Pyati P.V., Alter G.M., Rao N.S. (1990). Bacteriochlorophyll and Photosynthetic Reaction Centers in *Rhizobium* Strain BTAi 1. Appl. Environ. Microbiol..

[22] Giraud E., Fleischman D. (2004). Nitrogen-fixing symbiosis between photosynthetic bacteria and legumes. Photosynth. Res..

[23] Giraud E., Xu L., Chaintreuil C., Gargani D., Gully D., Sadowsky M.J. (2013). Photosynthetic *Bradyrhizobium* sp. strain ORS285 is capable of forming nitrogen-fixing root nodules on soybeans (Glycine max). Appl. Environ. Microbiol..

[24] Gevers D., Cohan F.M., Lawrence J.G., Spratt B.G., Coenye T., Feil E.J., Stackebrandt E., Van de Peer Y., Vandamme P., Thompson F.L. (2005). Opinion: Re-evaluating prokaryotic species. Nat. Rev. Microbiol..

[25] Kitahara K., Miyazaki K. (2013). Revisiting bacterial phylogeny: Natural and experimental evidence for horizontal gene transfer of 16S rRNA. Mob. Genet. Elem..

[26] Glaeser S.P., Kämpfer P. (2015). Multilocus sequence analysis (MLSA) in prokaryotic taxonomy. Syst. Appl. Microbiol..

[27] Aguilar O.M., López M.V., Riccillo P.M. (2001). The diversity of rhizobia nodulating beans in Northwest Argentina as a source of more efficient inoculant strains. J. Biotechnol..

[28] De Meyer S.E., De Beuf K., Vekeman B., Willems A. (2015). A large diversity of non-rhizobial endophytes found in legume root nodules in Flanders (Belgium). Soil Biol. Biochem..

[29] Gossmann J.A., Markmann K., Brachmann A., Rose L.E., Parniske M. (2012). Polymorphic infection and organogenesis patterns induced by a *Rhizobium leguminosarum* isolate from Lotus root nodules are determined by the host genotype. New Phytol..

[30] Ibáñez F., Angelini J., Taurian T., Tonelli M.L., Fabra A. (2009). Endophytic occupation of peanut root nodules by opportunistic Gammaproteobacteria. Syst. Appl. Microbiol..

[31] Kan F.L., Chen Z.Y., Wang E.T., Tian C.F., Sui X.H., Chen W.X. (2007). Characterization of symbiotic and endophytic bacteria isolated from root nodules of herbaceous legumes grown in Qinghai-Tibet plateau and in other zones of China. Arch. Microbiol..

[32] Muresu R., Polone E., Sulas L., Baldan B., Tondello A., Delogu G., Cappuccinelli P., Alberghini S., Benhizia Y., Benhizia H. (2008). Coexistence of predominantly nonculturable rhizobia with diverse, endophytic bacterial taxa within nodules of wild legumes. FEMS Microbiol. Ecol..

[33] Trujillo M.E., Alonso-Vega P., Rodríguez R., Carro L., Cerda E., Alonso P., Martínez-Molina E. (2010). The genus Micromonospora is widespread in legume root nodules: The example of *Lupinus angustifolius*. Isme J.

[34] Zgadzaj R., James E.K., Kelly S., Kawaharada Y., de Jonge N., Jensen D.B., Madsen L.H., Radutoiu S. (2015). A Legume Genetic Framework Controls Infection of Nodules by Symbiotic and Endophytic Bacteria. PLoS Genet..

[35] Mrabet M., Mnasri B., Romdhane S.B., Laguerre G., Aouani M.E., Mhamdi R. (2006). *Agrobacterium* strains isolated from root nodules of common bean specifically reduce nodulation by *Rhizobium gallicum*. Fems Microbiol. Ecol..

[36] Bai Y., Zhou X., Smith D.L. (2003). Enhanced Soybean Plant Growth Resulting from Coinoculation of Strains with. Crop Sci..

[37] Tokala R.K., Strap J.L., Jung C.M., Crawford D.L., Salove M.H., Deobald L.A., Bailey J.F., Morra M.J. (2002). Novel Plant-Microbe Rhizosphere Interaction Involving *Streptomyces lydicus* WYEC108 and the Pea Plant (Pisum sativum). Appl. Environ. Microbiol..

[38] Glick B.R. (1995). The enhancement of plant growth by free-living bacteria. Can. J. Microbiol..

[39] Datta B., Chakrabartty P.K. (2014). Siderophore biosynthesis genes of *Rhizobium* sp. isolated from Cicer arietinum L. 3 Biotech.

[40] Halder A.K., Mishra A.K., Bhattacharyya P., Chakrabartty P.K. (1990). Solubilization of rock phosphate by *Rhizobium* and *Bradyrhizobium*. J. Gen. Appl. Microbiol..

[41] Peix A., Rivas-Boyero A.A., Mateos P.F., Rodriguez-Barrueco C., Martínez-Molina E., Velazquez E. (2001). Growth promotion of chickpea and barley by a phosphate solubilizing strain of *Mesorhizobium mediterraneum* under growth chamber conditions. Soil Biol. Biochem..

[42] Datta C., Basu P.S. (2000). Indole acetic acid production by a *Rhizobium* species from root nodules of a leguminous shrub, Cajanus cajan. Microbiol. Res..

[43] Kudoyarova G.R., Melentiev A.I., Martynenko E.V., Timergalina L.N., Arkhipova T.N., Shendel G.V., Kuz’mina L.Y., Dodd I.C., Veselov S.Y. (2014). Cytokinin producing bacteria stimulate amino acid deposition by wheat roots. Plant Physiol. Biochem..

[44] Malik D.K., Sindhu S.S. (2011). Production of indole acetic acid by *Pseudomonas* sp.: Effect of coinoculation with *Mesorhizobium* sp. Cicer on nodulation and plant growth of chickpea (*Cicer arietinum*). Physiol. Mol. Biol. Plants.

[45] Chao W.-L. (1990). Antagonistic activity of *Rhizobium* spp. against beneficial and plant pathogenic fungi. Lett. Appl. Microbiol..

[46] Gómez Expósito R., Postma J., Raaijmakers J.M., De Bruijn I. (2015). Diversity and Activity of *Lysobacter* Species from Disease Suppressive Soils. Front. Microbiol..

[47] Ko H.-S., Jin R.-D., Krishnan H.B., Lee S.-B., Kim K.-Y. (2009). Biocontrol ability of *Lysobacter antibioticus* HS124 against *Phytophthora* blight is mediated by the production of 4-hydroxyphenylacetic acid and several lytic enzymes. Curr. Microbiol..

[48] Hahn N.J. (1966). The Congo red reaction in bacteria and its usefulness in the identification of rhizobia. Can. J. Microbiol..

[49] Somasegaran P., Hoben H.J. (1994). Handbook for Rhizobia: Methods in legume-rhizobium technology.

[50] Versalovic J., Koeuth T., Lupski J.R. (1991). Distribution of repetitive DNA sequences in eubacteria and application to fingerprinting of bacterial genomes. Nucleic Acids Res..

[51] Heras J., Domínguez C., Mata E., Pascual V., Lozano C., Torres C., Zarazaga M. (2015). GelJ--a tool for analyzing DNA fingerprint gel images. BMC Bioinform..

[52] Sneath P., Sokal R.R. (1973). Numerical Taxonomy: The Principles and Practice of Numerical Classification.

[53] Herrera-Cervera J.A., Caballero-Mellado J., Laguerre G., Tichy H.-V., Requena N., Amarger N., MartÃ-nez-Romero E., Olivares J., Sanjuan J. (1999). At least five rhizobial species nodulate *Phaseolus vulgaris* in a Spanish soil. FEMS Microbiol. Ecol..

[54] Weisburg W.G., Barns S.M., Pelletier D.A., Lane D.J. (1991). 16S ribosomal DNA amplification for phylogenetic study. J. Bacteriol..

[55] Gaunt M.W., Turner S.L., Rigottier-Gois L., Lloyd-Macgilp S.A., Young J.P. (2001). Phylogenies of *atp*D and *rec*A support the small subunit rRNA-based classification of rhizobia. Int. J. Syst. Evol. Microbiol..

[56] Stepkowski T., Czaplińska M., Miedzinska K., Moulin L. (2003). The variable part of the dnaK gene as an alternative marker for phylogenetic studies of rhizobia and related alpha Proteobacteria. Syst. Appl. Microbiol..

[57] Laguerre G., Nour S.M., Macheret V., Sanjuan J., Drouin P., Amarger N. (2001). Classification of rhizobia based on *nod*C and *nif*H gene analysis reveals a close phylogenetic relationship among *Phaseolus vulgaris* symbionts. Microbiology.

[58] Nguyen L.-T., Schmidt H.A., von Haeseler A., Minh B.Q. (2015). IQ-TREE: A fast and effective stochastic algorithm for estimating maximum-likelihood phylogenies. Mol. Biol. Evol..

[59] Towns J., Cockerill T., Dahan M., Foster I., Gaither K., Grimshaw A., Hazlewood V., Lathrop S., Lifka D., Peterson G.D. (2014). XSEDE: Accelerating Scientific Discovery. Comput. Sci. Eng..

[60] Miller M.A., Pfeiffer W., Schwartz T. (2010). Creating the CIPRES Science Gateway for inference of large phylogenetic trees. Proceedings of the 2010 Gateway Computing Environments Workshop (GCE).

[61] Kalyaanamoorthy S., Minh B.Q., Wong T.K.F., von Haeseler A., Jermiin L.S. (2017). ModelFinder: Fast model selection for accurate phylogenetic estimates. Nat. Methods.

[62] Hoang D.T., Chernomor O., von Haeseler A., Minh B.Q., Vinh L.S. (2018). UFBoot2: Improving the Ultrafast Bootstrap Approximation. Mol. Biol. Evol..

[63] Letunic I., Bork P. (2019). Interactive Tree Of Life (iTOL) v4: Recent updates and new developments. Nucleic Acids Res..

[64] Jensen H.L. (1941). Nitrogen fixation in leguminous plants. I. General characters of root-nodule bacteria isolated from species of *Medicago* and *Trifolium* in Australia. Proc. Linn. Soc. New South Wales.

[65] Ferreira E.M., Marques J.F. (1992). Selection of Portuguese *Rhizobium leguminosarum* bv. *trifolii* strains for production of legume inoculants. Plant Soil.

[66] Illmer P., Barbato A., Schinner F. (1995). Solubilization of hardly-soluble AlPO4 with P-solubilizing microorganisms. Soil Biol. Biochem..

[67] Beringer J.E. (1974). R factor transfer in Rhizobium leguminosarum. J. Gen. Microbiol..

[68] Pérez-Miranda S., Cabirol N., George-Téllez R., Zamudio-Rivera L.S., Fernández F.J. (2007). O-CAS, a fast and universal method for siderophore detection. J. Microbiol. Methods.

[69] Verma S.C., Ladha J.K., Tripathi A.K. (2001). Evaluation of plant growth promoting and colonization ability of endophytic diazotrophs from deep water rice. J. Biotechnol..

[70] Brown J.R., Masuchi Y., Robb F.T., Doolittle W.F. (1994). Evolutionary relationships of bacterial and archaeal glutamine synthetase genes. J. Mol. Evol..

[71] Stępkowski T., Zak M., Moulin L., Króliczak J., Golińska B., Narożna D., Safronova V.I., Mądrzak C.J. (2011). *Bradyrhizobium canariense* and *Bradyrhizobium japonicum* are the two dominant rhizobium species in root nodules of lupin and serradella plants growing in Europe. Syst. Appl. Microbiol..

[72] Vinuesa P., Silva C., Werner D., Martínez-Romero E. (2005). Population genetics and phylogenetic inference in bacterial molecular systematics: The roles of migration and recombination in *Bradyrhizobium* species cohesion and delineation. Mol. Phylogenet. Evol..

[73] Bourebaba Y., Durán D., Boulila F., Ahnia H., Boulila A., Temprano F., Palacios J.M., Imperial J., Ruiz-Argüeso T., Rey L. (2016). Diversity of *Bradyrhizobium* strains nodulating *Lupinus micranthus* on both sides of the Western Mediterranean: Algeria and Spain. Syst. Appl. Microbiol..

[74] Mousavi S.A., Willems A., Nesme X., de Lajudie P., Lindström K. (2015). Revised phylogeny of *Rhizobiaceae*: Proposal of the delineation of *Pararhizobium* gen. nov., and 13 new species combinations. Syst. Appl. Microbiol..

[75] Park J.H., Kim R., Aslam Z., Jeon C.O., Chung Y.R. (2008). *Lysobacter capsici* sp. nov., with antimicrobial activity, isolated from the rhizosphere of pepper, and emended description of the genus *Lysobacter*. Int. J. Syst. Evol. Microbiol..

[76] Puopolo G., Sonego P., Engelen K., Pertot I. (2014). Draft Genome Sequence of *Lysobacter capsici* AZ78, a Bacterium Antagonistic to Plant-Pathogenic Oomycetes. Genome Announc..

[77] Lin H., Hu S., Liu R., Chen P., Ge C., Zhu B., Guo L. (2016). Genome Sequence of *Pseudomonas koreensis* CRS05-R5, an Antagonistic Bacterium Isolated from Rice Paddy Field. Front. Microbiol.

[78] Rafikova G.F., Korshunova T.Y., Minnebaev L.F., Chetverikov S.P., Loginov O.N. (2016). A new bacterial strain, Pseudomonas koreensis IB-4, as a promising agent for plant pathogen biological control. Microbiology.

[79] Ganeshan G., Kumar A.M. (2005). *Pseudomonas fluorescens*, a potential bacterial antagonist to control plant diseases. J. Plant Interact..

[80] Trapet P., Avoscan L., Klinguer A., Pateyron S., Citerne S., Chervin C., Mazurier S., Lemanceau P., Wendehenne D., Besson-Bard A. (2016). The *Pseudomonas fluorescens* Siderophore Pyoverdine Weakens *Arabidopsis thaliana* Defense in Favor of Growth in Iron-Deficient Conditions. Plant Physiol..

[81] Lewis R.W., Islam A., Opdahl L., Davenport J.R., Sullivan T.S. (2019). Comparative Genomics, Siderophore Production, and Iron Scavenging Potential of Root Zone Soil Bacteria Isolated from ‘Concord’ Grape Vineyards. Microb. Ecol..

[82] Gusain Y.S., Kamal R., Mehta C.M., Singh U.S., Sharma A.K. (2015). Phosphate solubilizing and indole-3-acetic acid producing bacteria from the soil of Garhwal Himalaya aimed to improve the growth of rice. J. Env. Biol..

[83] Kasotia A., Choudhary D.K. (2016). Induced Inorganic Phosphate Solubilization Through N-Methyl-N´-Nitro-N-Nitrosoguanidine Treated Mutants of *Pseudomonas koreensis* Strain AK-1 (MTCC Number 12058) under Polyethylene Glycol. Proc. Natl. Acad. Sci. USA.

[84] Kwon S.W., Kim J.S., Park I.C., Yoon S.H., Park D.H., Lim C.K., Go S.J. (2003). *Pseudomonas koreensis* sp. nov., *Pseudomonas umsongensis* sp. nov. and *Pseudomonas jinjuensis* sp. nov., novel species from farm soils in Korea. Int. J. Syst. Evol. Microbiol..

[85] Daimon H., Nobuta K., Ohe M., Harada J., Nakayama Y. (2006). Tricalcium Phosphate Solubilization by Root Nodule Bacteria of *Sesbania cannabina* and *Crotalaria juncea*. Plant Prod. Sci..

[86] Das K., Prasanna R., Saxena A.K. (2017). Rhizobia: A potential biocontrol agent for soil borne fungal pathogens. Folia Microbiol. (Praha).

[87] Deryło M., Choma A., Puchalski B., Suchanek W. (1994). Siderophore activity in *Rhizobium* species isolated from different legumes. Acta Biochim. Pol..

[88] Afzal A., Bano A. (2008). *Rhizobium* and Phosphate Solubilizing Bacteria Improve the Yield and Phosphorus Uptake in Wheat (*Triticum aestivum*). Int. J. Agric. Biol..

[89] Ahmad M., Zahir Z.A., Khalid M., Nazli F., Arshad M. (2013). Efficacy of *Rhizobium* and *Pseudomonas* strains to improve physiology, ionic balance and quality of mung bean under salt-affected conditions on farmer’s fields. Plant Physiol. Biochem..

[90] Egamberdieva D., Berg G., Lindström K., Räsänen L.A. (2010). Co-inoculation of *Pseudomonas* spp. with *Rhizobium* improves growth and symbiotic performance of fodder galega (*Galega orientalis* Lam.). Eur. J. Soil Biol..

[91] Videira e Castro I., de Castro Silva M., Fernandez C., Colavolpe B., Machado H., Zúñiga-Dávila D., González-Andrés F., Ormeño-Orrillo E. (2019). The Potential of Nitrogen-Fixing Bacteria in the Sustainability of Agro-Forestry Ecosystems. Microbial Probiotics for Agricultural Systems: Advances in Agronomic Use.

[92] Pandya M., Rajput M., Rajkumar S. (2015). Exploring plant growth promoting potential of non rhizobial root nodules endophytes of *Vigna radiata*. Microbiology.

[93] Saini R., Dudeja S.S., Giri R., Kumar V. (2015). Isolation, characterization, and evaluation of bacterial root and nodule endophytes from chickpea cultivated in Northern India. J. Basic Microbiol..

[94] Zhao L., Xu Y., Lai X. (2017). Antagonistic endophytic bacteria associated with nodules of soybean (*Glycine max* L.) and plant growth-promoting properties. Braz. J. Microbiol..

[95] Cardoso P., Alves A., Silveira P., Sá C., Fidalgo C., Freitas R., Figueira E. (2018). Bacteria from nodules of wild legume species: Phylogenetic diversity, plant growth promotion abilities and osmotolerance. Sci. Total Environ..

[96] Ampomah O.Y., Huss-Danell K. (2011). Genetic diversity of root nodule bacteria nodulating Lotus corniculatus and Anthyllis vulneraria in Sweden. Syst. Appl. Microbiol..

[97] Sánchez M., Ramírez-Bahena M.-H., Peix A., Lorite M.J., Sanjuán J., Velázquez E., Monza J. (2014). *Phyllobacterium loti* sp. nov. isolated from nodules of Lotus corniculatus. Int. J. Syst. Evol. Microbiol..

