# Supplementary material for: Diversity, Phylogeny and Plant Growth Promotion Traits of Nodule Associated Bacteria Isolated from Lotus parviflorus
Source: Microorganisms. 2020 Mar 31;8(4):499. doi: 10.3390/microorganisms8040499 (PMC7232477; doi:10.3390/microorganisms8040499)
Supplement: Supplementary file 1 [file microorganisms-08-00499-s001.pdf]

Supplementary materials

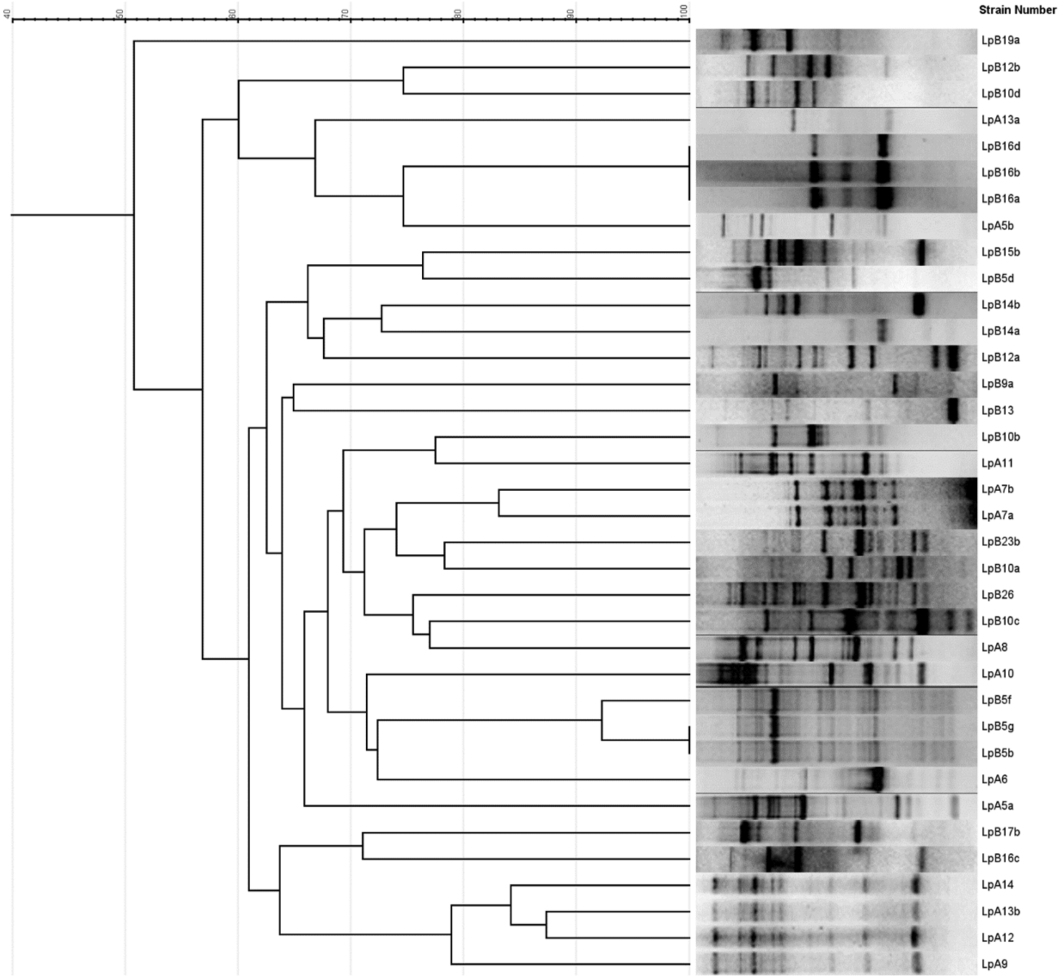

Figure S1. UPGMA dendrogram of REP-PCR genomic fingerprints from the bacterial.isolates.

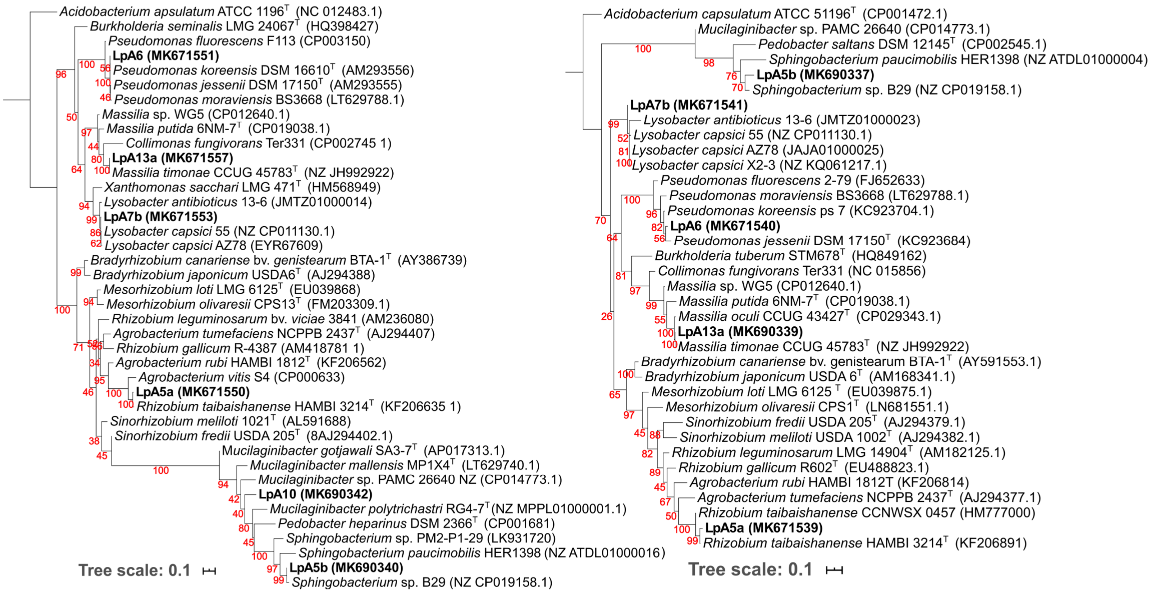

Figure S2. Phylogeny of *atp*D (left) and *rec*A (right) genes based on 468 and 329 positions, respectively. Analyses were conducted with IQ-tree using Maximum Likelihood (ML) and ModelFinder (Best model: TIMe+I+G4 (*atp*D) and TIM+F+I+G4 (*rec*A)) and methods. Confidence levels of bootstrap are presented in red near each node. NCBI Genebank accession codes are present next to each strain. *Acidobacterium capsulatum* 1372^{T} (NC_012483.1 and CP001472.1), a bacterium from the Acidobacteria phylum, was selected as an outgroup. Isolates obtained from *L. parviflorus* are in bold.

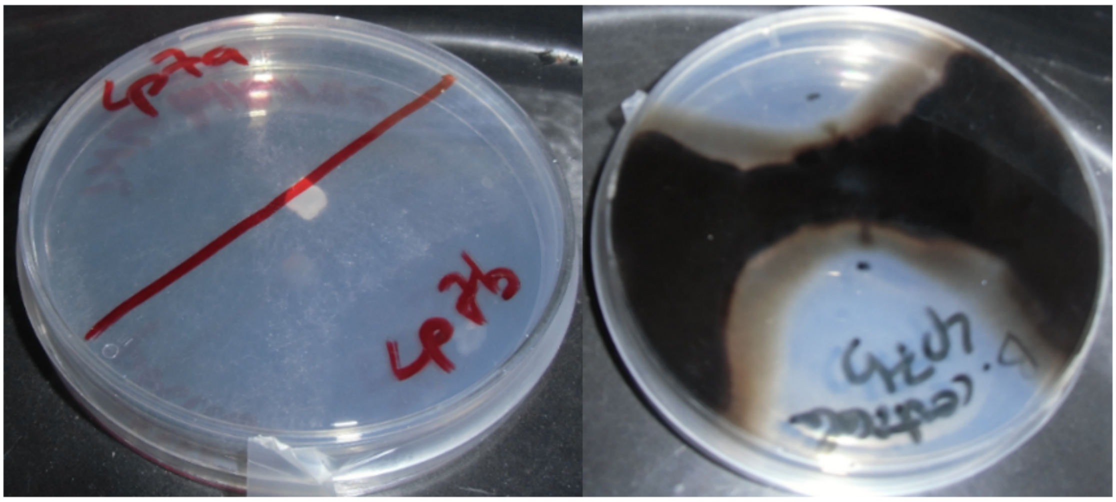

Figure S3. Inhibition of *P. cinnamomi* (left) and *B. corticola* (right) growth by the antagonistic activity of isolate LpA7b. In this figure Lp7a and Lp7b represent LpA7a and LpA7b.

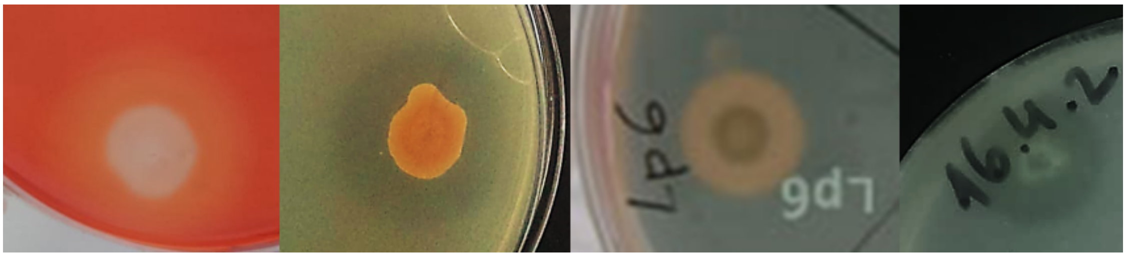

(a) **(b)** **(c)** **(d)**

Figure S4. *In vitro* PGPR activities showed. **(a)** and **(b)** Hydrolytic enzyme production, cellulases (**a**; isolate LpB9b) and pectinases (**b**; isolate LpB9b); **(c)** Siderophore production (isolate LpA6); **(d)** P solubilization (isolate LpB16d). In this figure Lp6 and 16.4.2 represent LpA6 and LpB16d.
